# Meta-Analysis of Image-Based Versus Probe-Based Parathyroid Near-Infrared Autofluorescence

**DOI:** 10.7759/cureus.80565

**Published:** 2025-03-14

**Authors:** Mackenzie J Reece, Travis W Stevenson, Margaret Liederbach, Sarah Yu, Sameep Kadakia, Mohamedkazim M Alwani

**Affiliations:** 1 Department of Surgery, Wright State University Boonshoft School of Medicine, Dayton, USA; 2 Department of Otolaryngology-Head and Neck Surgery, University Hospitals and Case Western Reserve University, Cleveland, USA; 3 Department of Otolaryngology-Head and Neck Surgery, Premier Health, Dayton, USA

**Keywords:** autofluorescence, meta-analysis, near-infrared, niraf, parathyroid surgery, systematic review, thyroid surgery

## Abstract

Proper identification of parathyroid tissue is a critical component in surgery involving the thyroid and parathyroid gland (PG). In recent years, near infrared autofluorescence (NIRAF) has been investigated as a non-invasive strategy to detect PG in parathyroidectomy and in PG preservation in thyroidectomy. There are currently two FDA approved NIRAF modalities, image-based and probe-based. The aim of this meta-analysis is to evaluate the efficacy of these two NIRAF modalities. PubMed, Scopus, and MEDLINE were utilized, with 238 studies analyzed via independent, blinded review. Studies from January 2000 to February 2023, Boolean phrase “parathyroid autofluorescence”, written in English, and included results found within the body of the article were the inclusion criteria used. Conference abstracts, reviews, case reports, commentary, discussion and letter, non-English, animal studies, in vitro studies, contrast enhanced fluorescence, and NIRAF with use of indocyanine green, were the exclusion criteria used. Five studies were enrolled based on inclusion and exclusion criteria. The estimated overall accuracy of image-based methods is 0.96 (95% CI of (0.87, 0.99)), while the estimated overall accuracy of probe-based methods is 0.93 (95% CI of (0.92, 0.94)). With p=0.36, there is insufficient evidence to indicate a significant difference in overall accuracy, sensitivity and specificity between image-based methods and probe-based methods. Both imaging and probe-based detection modalities offer effective, noninvasive means for identifying parathyroid glands intraoperatively. Further studies comparing the efficacy of these two modalities are needed to further differentiate their clinical performance.

## Introduction and background

Proper identification of parathyroid tissue is a critical component in surgery involving the thyroid and parathyroid gland (PG). The PG produces parathyroid hormone (PTH) which serves as a primary regulator of the body’s total calcium levels by increasing serum calcium via increased renal and enteric reabsorption [1,2]. PTH simultaneously decreases total body phosphate via increased excretion and decreased reabsorption [1-3]. The balance between these ions is important for cellular metabolism and bone maintenance. Depletion of PTH due to hypoparathyroidism, such as in the case of incidental removal during surgery, can lead to severe, long-term outcomes for patients including osteoporosis and neurological dysfunction [4,5]. The PG’s small size and close relationship with the surrounding fat of the thyroid gland can make its preservation a challenge. Postsurgical hypoparathyroidism (PH) is a common complication following thyroidectomy and parathyroidectomy which results in decreased circulating PTH and subsequent hypocalcemia and hyperphosphatemia [6]. Even for the experienced surgeon, PG preservation and identification can be challenging. 

Traditional methods of PG identification include preoperative imaging such as cervical ultrasonography and 99mTC-MIBI scintigraphy combined with intraoperative surgeon experience to recognize PGs [7-9]. In recent years, magnetic resonance imaging (MRI), positron emissions tomography (PET), and parathyroid four-dimensional computed tomography (4D-CT) have been utilized in pre-operative identification of parathyroid adenomas. However, PH remains a prevalent complication. In the United States, a study in 2014 by Powers et al. (2013) found the prevalence of PH to be 32 per 100,000 [10]. This could be explained, in part, by the fact that these imaging techniques are often more accurate in identifying abnormal PGs, but are less sensitive to normal PGs. Intra-operative methods of PG identification such as Raman spectroscopy, indocyanine green (ICG), and near-infrared autofluorescence (NIRAF) have become more commonly used and have been shown to be more accurate in localizing PGs [7,10-12].

ICG angiography has been a safe and effective tool as a contrast enhancing dye since it was first approved for clinical use in the late 1950s. In relation to PG identification, it has been a useful intraoperative tool because it can be readily absorbed by both normal and abnormal PGs [13,14]. However, one of the major limitations of ICG is that it is non-specific for PGs and can also be readily taken up by the thyroid and surrounding tissue, making a clear distinction of the PG difficult [15,16]. In turn, autofluorescence has become a promising intraoperative method due to its ability to target electromagnetic properties specific to the PG. Relative to the other surrounding tissues in the neck, the PG demonstrates a strong NIRAF [12,17-19]. This could be due to the presence of potential natural fluorophores like calcium-sensing receptors (CaSR) and vitamin D, yet this has not been proven [18,19]. When exposed to NIR, the PG emits light at approximately 820-830 nm which is roughly two to 20 times greater than the thyroid gland and surrounding tissue [19,20]. In contrast to ICG, NIRAF is a rapid, non-invasive, and label-free method of PG identification. Currently, there are two FDA approved versions of NIRAF in the United States: probe-based and image-based.

Image-based NIRAF typically requires no contact and relies on light emission collection from a handheld camera [20-22]. The illuminated tissue is then displayed on a display monitor for the surgeon to visualize. Additionally, this modality may be used with ICG to determine the viability of blood flow to the PG. Though hands free, this method does not provide quantitative information regarding the autofluorescence of surrounding tissues; thus, this method is more dependent on the surgeon to subjectively determine relative fluorescence of the tissues. On the other hand, probe-based NIRAF requires the use of a handheld probe that must be in direct contact with the tissue [21,22]. Several studies have shown that this method has very high sensitivity in identifying PGs and can be used in bright rooms as opposed to image-based with ICG [23,24]. However, a potential disadvantage of this modality is that it requires the surgeon to surgically expose the suspected tissue for analysis and cannot be used to assess blood supply as opposed to image-based NIRAF [23,24]. Currently, the two FDA approved NIRAF systems for clinical use in the United States are an image-based system by Fluobeam (Inomed, Emmendingen, Germany), and a probe-based system by PTeye (Medtronics, Minneapolis, MN) [20-24]. Each of these systems has been shown to display high accuracy, but the comparison of the two has not been thoroughly studied. The aim of this meta-analysis is to evaluate if there is a difference in efficacy between these two NIRAF modalities.

## Review

Methods

Search Strategy and Selection Criteria

A comprehensive systematic literature review was conducted searching PubMed, Scopus, and MEDLINE, yielding 238 publications, from which five studies were enrolled following independent, blinded review. One reviewer (MJL) performed the initial database search and screened the results for duplicates. Three blinded reviewers (MJL, MJR, TWS) screened the remaining studies by title and abstract according to defined inclusion and exclusion criteria. Inclusion criteria used were studies from January 2000 to February 2023, Boolean phrase “parathyroid autofluorescence”, written in English, and included results found within the body of the article. Exclusion criteria used were conference abstracts, reviews, case reports, commentary, discussion and letter, non-English, animal studies, in vitro studies, contrast enhanced fluorescence, and NIRAF with use of ICG. 

Discrepancies between the final selections were reviewed and resolved by joint decision by the reviewing authors. Three reviewers (MJL, MJR, TWS) extracted relevant data from the selected studies and compiled the results in a Microsoft Excel spreadsheet. Citations were uploaded to Mendeley reference manager and further exported to Rayyan systematic review software. This systematic review was conducted according to the principles and recommendations of the Preferred Reporting Items for Systematic Reviews and Meta-Analyses (PRISMA) statement [25,26].

**Figure 1:**
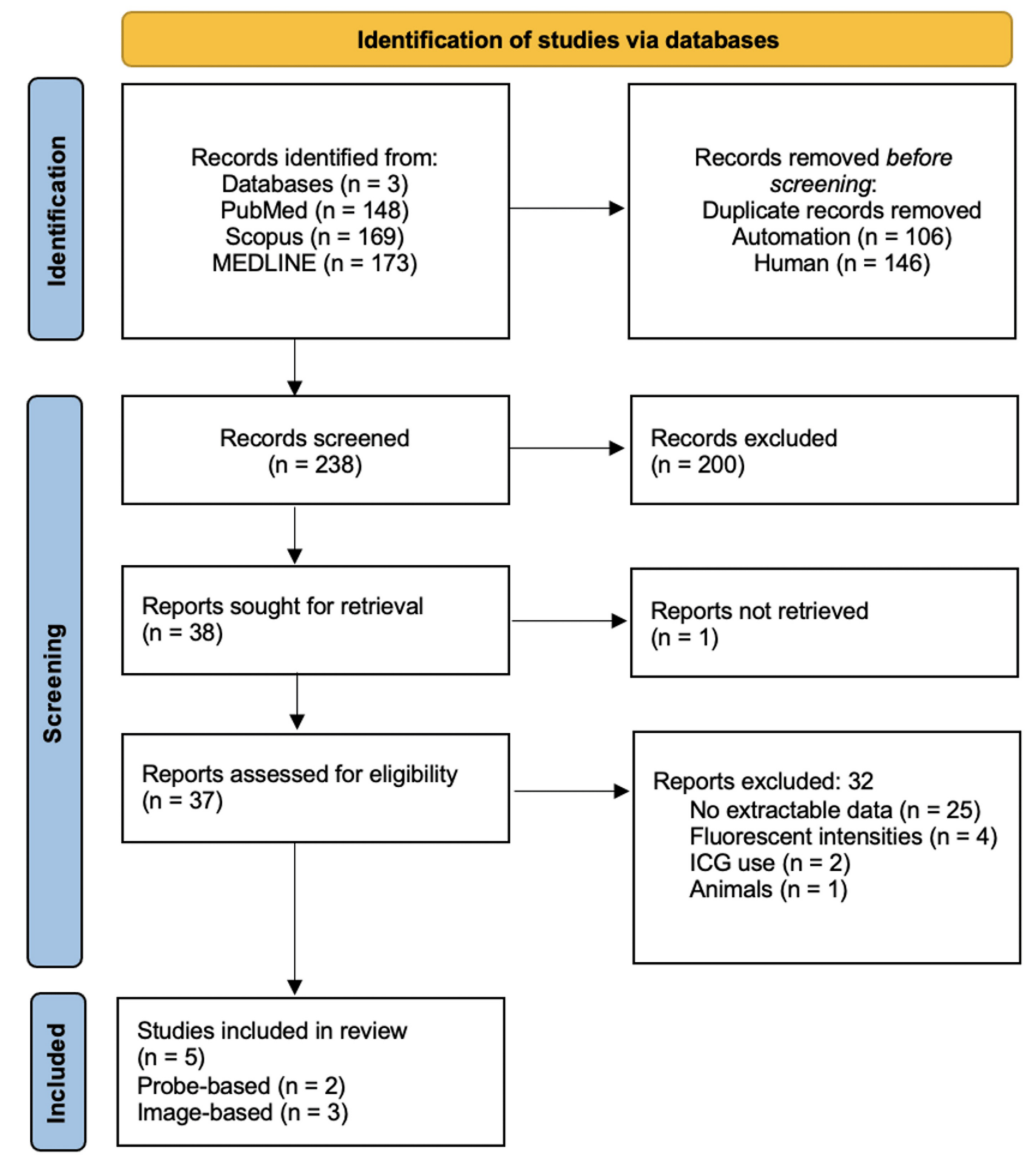
PRISMA flow chart of study selection. PRISMA, Preferred Reporting Items for Systematic Reviews and Meta-Analyses.

Of the five studies enrolled, two were probe-based with 250 subjects, and three were image-based with 368 subjects. Figure 1 represents the study selection process. The primary outcomes compared device performance according to their accuracy, sensitivity, and specificity. Secondary outcomes included negative and positive predictive values. 

Data Analysis

The “meta” package in RStudio (v4.1.0; R Core Team 2023) was used for all analyses [27]. A random effects model was assumed to account for possible differences in results based on external factors specific to a given study, such as the experience level of the surgeon or patient demographics. The two methods (image or probe) were treated as subgroups. Logit transformations were used to calculate overall proportions, 95% confidence intervals (CIs) were created via the Clopper-Pearson method, and a level of significance of α = 0.05 was used throughout to assess statistical significance.

Results

Table 1 summarizes the basic information of the five included eligible studies [28-31]. The studies were published between 2018 and 2021. All were prospective, four conducted in the United States and one in South Korea. Two utilized the probe-based technology for a total of 250 patients. Three utilized the image-based for a total of 368 patients. Note that Thomas et al. (2019) used both image-based and probe-based methods [30]. However, the image-based method was always performed first, so only the image-based results were included in the study, given the probe-based results were susceptible to bias from being performed second. Accuracy, specificity, sensitivity, positive predictive value (PPV), and negative predictive value (NPV) were compared between the two modalities. 

**Table 1:** Characteristics of included studies. PDE, photodynamic eye.

Study ID	First author	Publication time	Type of study	Country/region	Instrument	Patients
1	Thomas et al. [30]	2019	Prospective	America	PDE-Neo II, PTeye	20
2	Kose et al. [31]	2020	Prospective	America	Fluobeam	310
3	Kim et al. [27]	2018	Prospective	South Korea	Fluorescence imaging	38
4	Kiernan et al. [28]	2021	Prospective	America	PTeye	83
5	Thomas et al. [29]	2021	Prospective	America	PTeye	167

A true positive (TP) is defined as correctly identifying and preserving PG during parathyroid preservation or identifying and removing a PG during parathyroidectomy. A true negative (TN) is defined as correctly identifying non-PG within the tissue specimen following thyroidectomy and the tissue specimen in question during parathyroidectomy. A false positive (FP) is defined as incorrectly identifying non-PG as PG during parathyroid preservation or incorrectly identifying non-PG as PG during parathyroidectomy. A false negative (FN) is defined as incorrectly identifying PG as non-PG during parathyroid preservation or incorrectly identifying non-PG as PG during parathyroidectomy.

**Figure 2:**
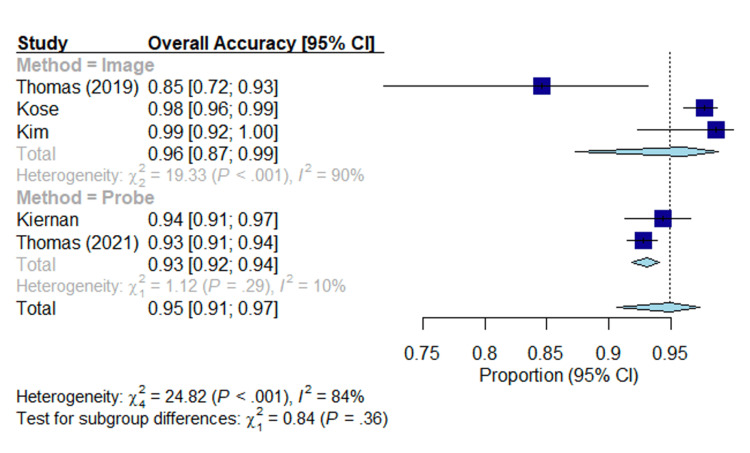
Forest plot; overall accuracy. Overall accuracy of each modality in each study. Lines represent confidence intervals. The dotted black line represents the mean of all study results. I2 value determines the level of heterogeneity. Kim et al. (2018) [27], Kiernan et al. (2021) [28], Thomas et al. (2021) [29], Thomas et al. (2019) [30], Kose et al. (2020) [31].

As shown in Figure 2, the overall accuracy is given in the forest plot. The estimated overall accuracy of image-based methods is 0.96 (95% CI of (0.87, 0.99)), while the estimated overall accuracy of probe-based methods is 0.93 (95% CI of (0.92, 0.94)). Based on a p-value of 0.36, there is not sufficient evidence to suggest there is a significant difference in overall accuracy between image-based methods and probe-based methods.

**Figure 3:**
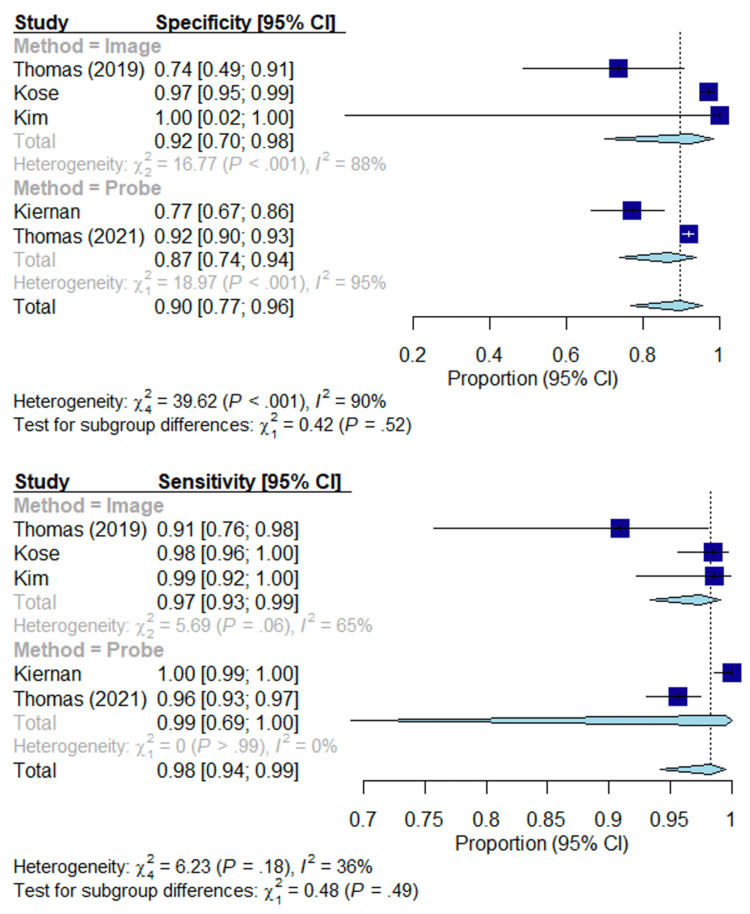
Forest plot; specificity and sensitivity. Overall specificity and sensitivity of each modality. Lines represent confidence intervals. The dotted black line represents the mean of all study results. I2 value determines the level of heterogeneity. Kim et al. (2018) [27], Kiernan et al. (2021) [28], Thomas et al. (2021) [29], Thomas et al. (2019) [30], Kose et al. (2020) [31].

**Figure 4:**
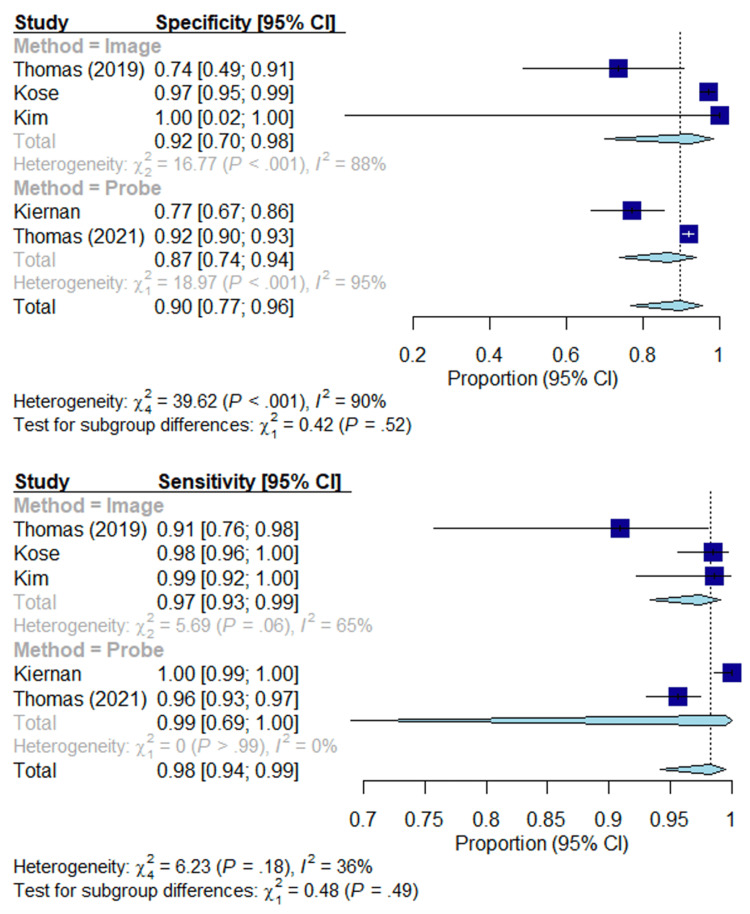
Positive predictive value (PPV) and negative predictive value (NPV). Overall PPV and NPV of each modality. Lines represent confidence intervals. The dotted black line represents the mean of all study results. I2 value determines the level of heterogeneity. Kim et al. (2018) [27], Kiernan et al. (2021) [28], Thomas et al. (2021) [29], Thomas et al. (2019) [30], Kose et al. (2020) [31].

As shown in Figures 3-4, the sensitivity, specificity, NPV, and PPV are given in the forest plots. The estimated sensitivity of image-based methods and probe-based methods were high (0.97, 0.99; p = 0.49) without sufficient evidence to suggest there is a significant difference in sensitivity between image-based methods and probe-based methods. The estimated specificity of image-based methods and probe-based methods were high (0.92, 0.87; p = 0.52) without sufficient evidence to suggest there is a significant difference. The estimated PPV of image-based methods and probe-based methods were high (0.96, 0.87; p = 0.17) without sufficient evidence to suggest there is a significant difference in PPV. The estimated NPV of image-based methods and probe-based methods were high (0.93, 0.99; p = 0.14) without a significant difference in NPV. 

**Figure 5:**
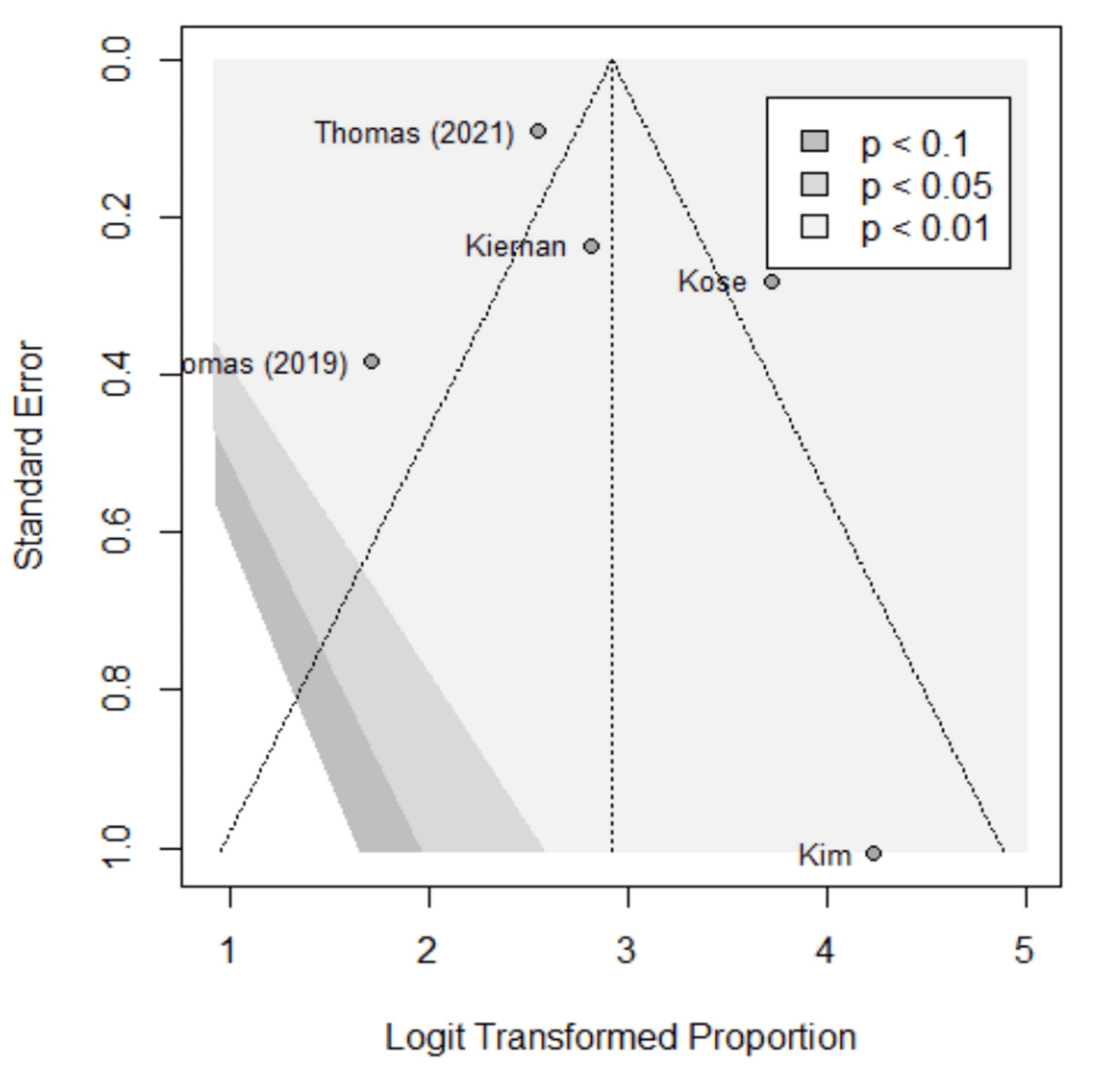
Funnel plot; accuracy. Funnel plot of the meta-analysis comparing accuracy of image-based and probe-based approaches. Kim et al. (2018) [27], Kiernan et al. (2021) [28], Thomas et al. (2021) [29], Thomas et al. (2019) [30], Kose et al. (2020) [31].

**Figure 6:**
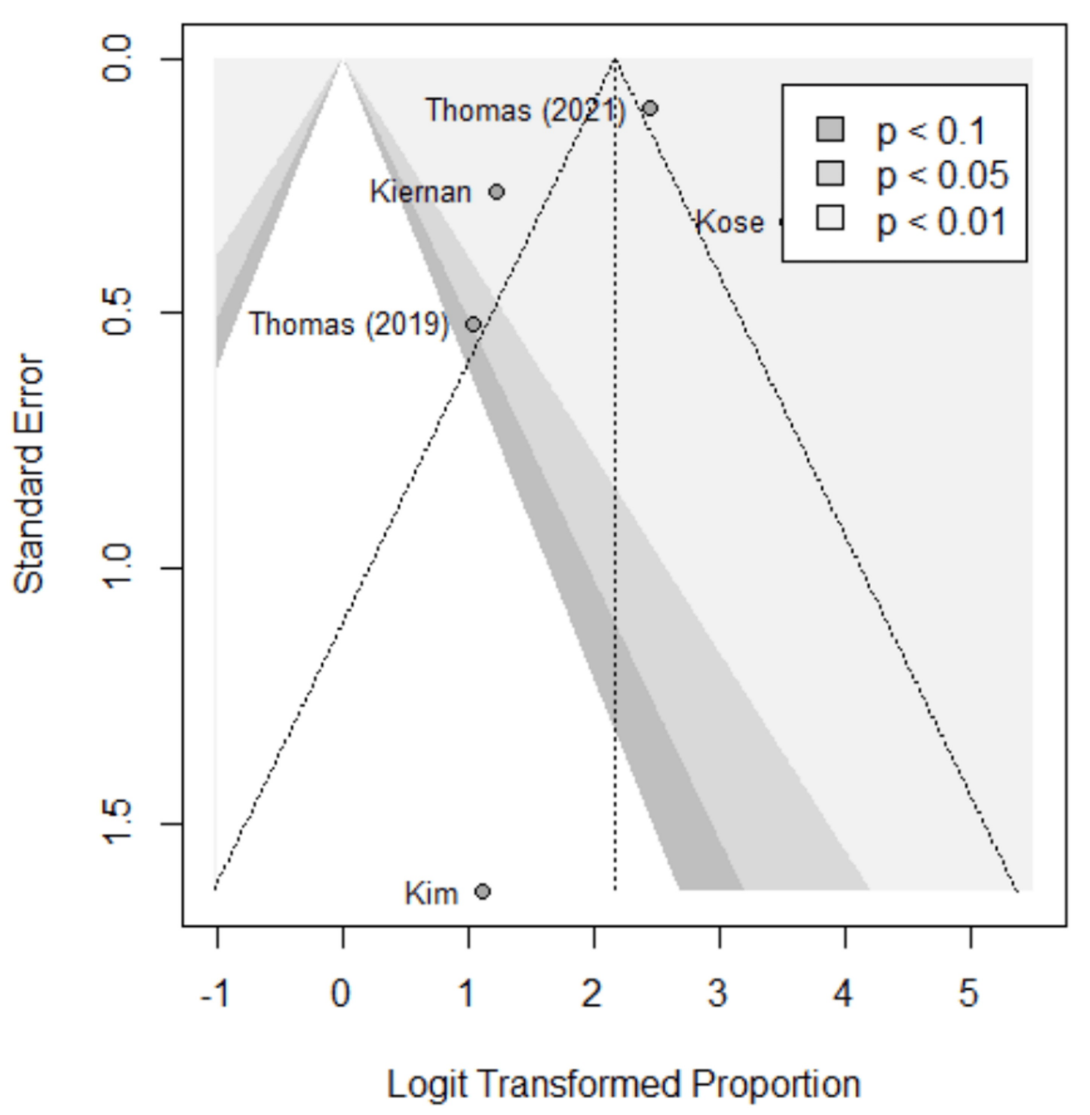
Funnel plot; specificity. Funnel plot of the meta-analysis comparing specificity of image-based and probe-based approaches. Kim et al. (2018) [27], Kiernan et al. (2021) [28], Thomas et al. (2021) [29], Thomas et al. (2019) [30], Kose et al. (2020) [31].

**Figure 7:**
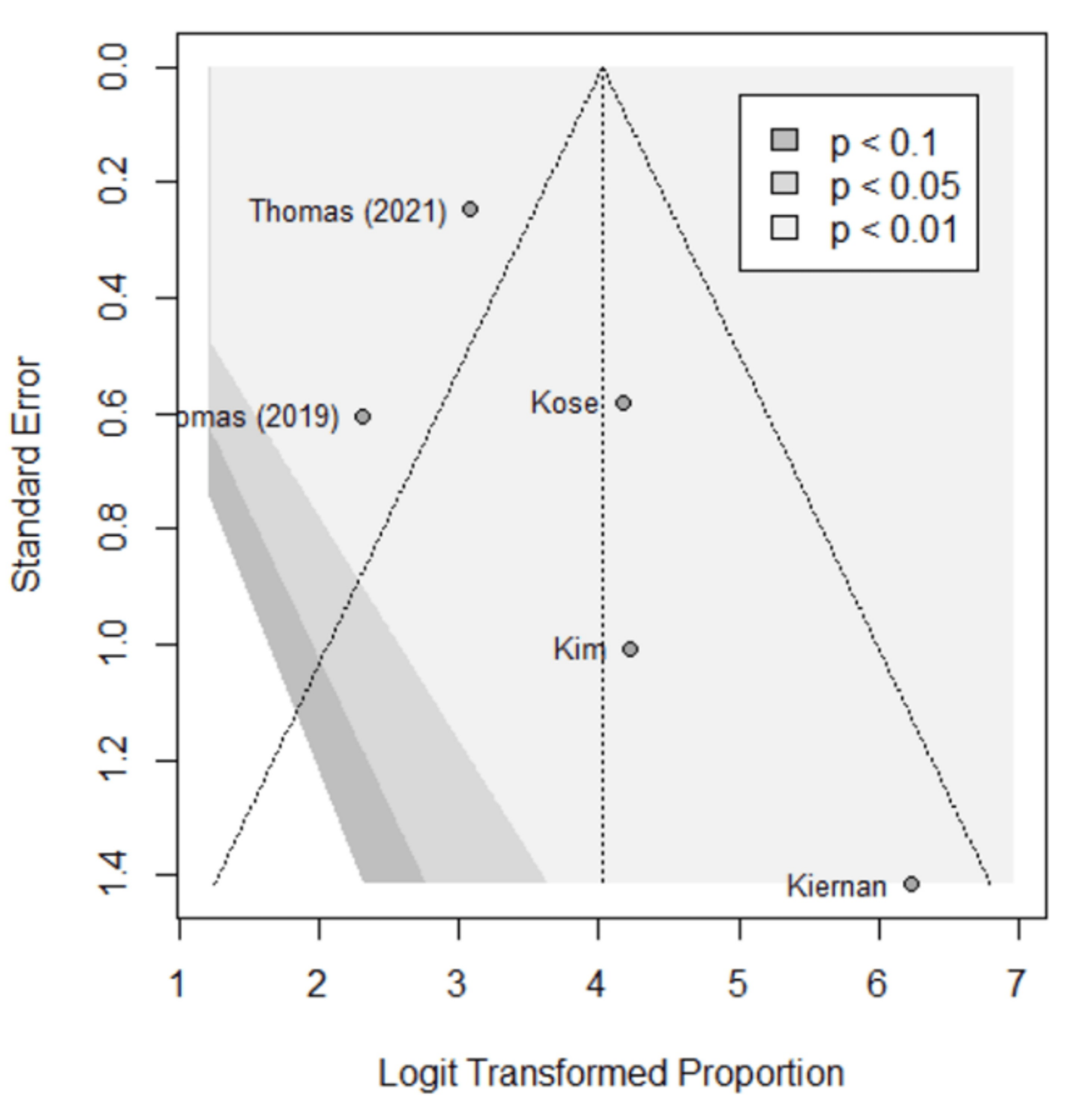
Funnel plot; sensitivity. Funnel plot of the meta-analysis comparing sensitivity of image-based and probe-based approaches. Kim et al. (2018) [27], Kiernan et al. (2021) [28], Thomas et al. (2021) [29], Thomas et al. (2019) [30], Kose et al. (2020) [31].

**Figure 8:**
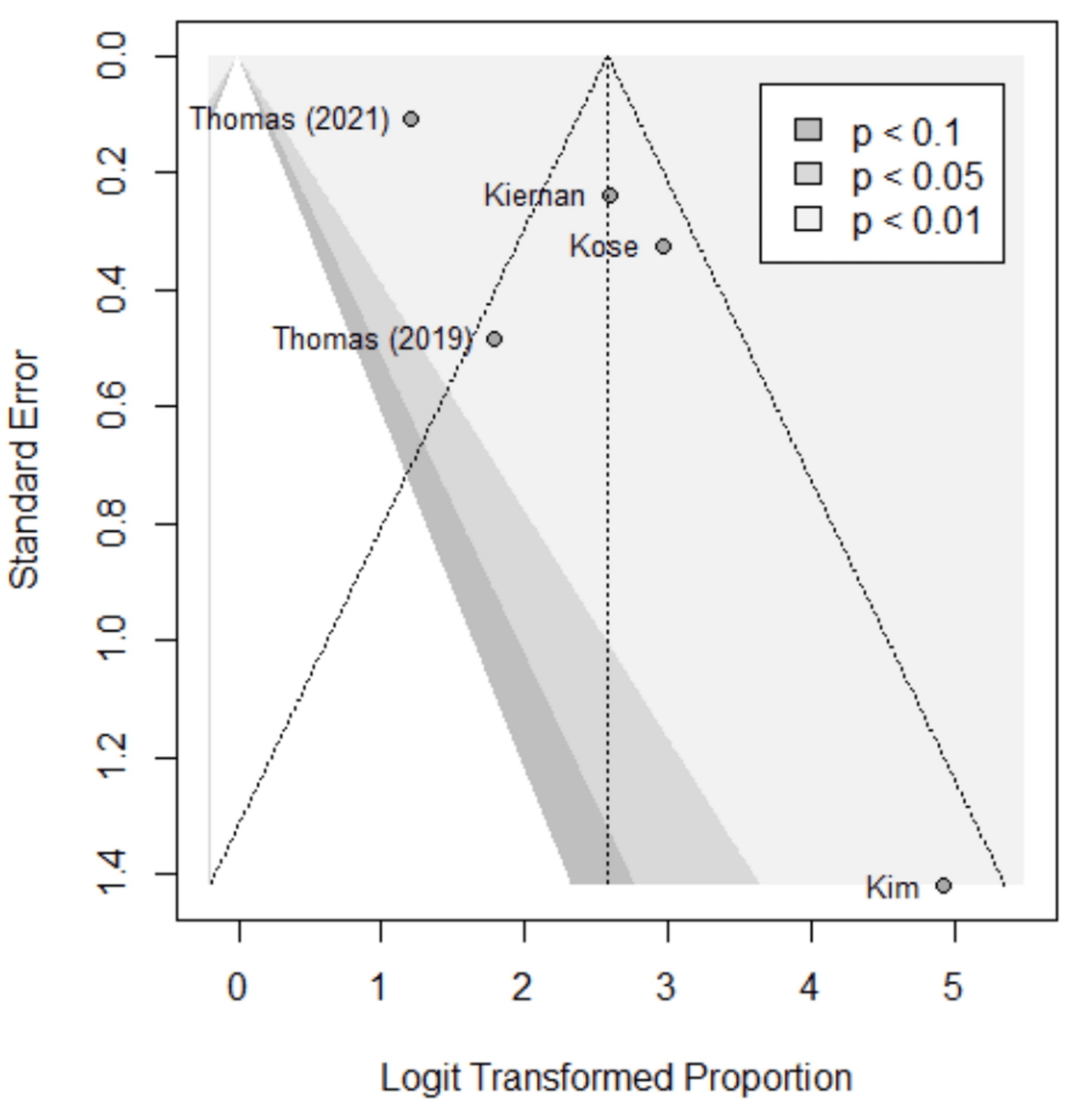
Funnel plot; positive predictive value (PPV). Funnel plot of the meta-analysis comparing PPV of image-based and probe-based approaches. Kim et al. (2018) [27], Kiernan et al. (2021) [28], Thomas et al. (2021) [29], Thomas et al. (2019) [30], Kose et al. (2020) [31].

**Figure 9:**
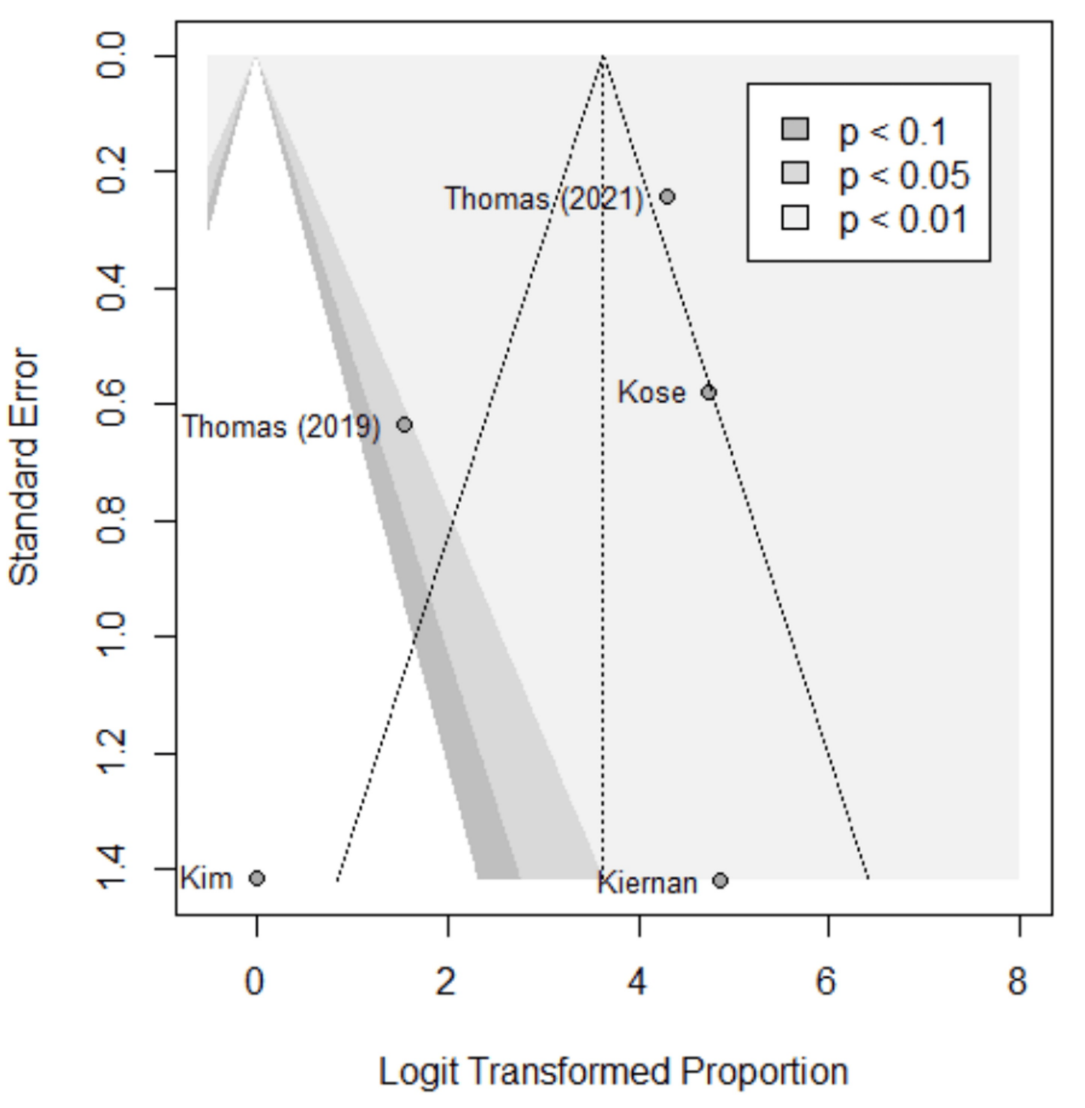
Funnel plot; negative predictive value (NPV). Funnel plot of the meta-analysis comparing NPV of image-based and probe-based approaches. Kim et al. (2018) [27], Kiernan et al. (2021) [28], Thomas et al. (2021) [29], Thomas et al. (2019) [30], Kose et al. (2020) [31].

Funnel plots were used to assess for publication bias, shown in Figures 5-9. Studies represented with lower sample sizes were noted to have larger standard errors, represented by Kim et al. (2018) and Kiernan et al. (2021) [27,28]. Publication bias is difficult to assess in this meta-analysis due to only five studies included and no significant difference found between the two methods.

Discussion

This systematic review and meta-analysis is the first to compare image-based and probe-based approaches based on the recent literature leading up to February 2023. Five studies included reporting the efficacy of image-based and probe-based approaches in detecting the near-infrared auto fluorescent properties of parathyroid tissue [27-31]. From the data available and the studies analyzed, there was no statistically significant difference in sensitivity, specificity, PPV, NPV, or overall accuracy between the two methods. However, this meta-analysis does demonstrate an overall high accuracy of both the probe and image-based approaches. Kim et al. (2018) reported a 100% accuracy rate using the probe-based method across 64 samples, 92.8% being identified before visualization by the surgeon [27]. Kiernan et al. (2021) reported a 94.3% accuracy using the probe-based imaging across a similar sample size [28]. Thomas et al. (2019) found an 84.6% accuracy using the image-based approach [30]. Later, Thomas et al. (2021) also found a 92.3% accuracy using the probe-based approach; however, this study was done with a smaller sample size [29]. Conversely, Kose et al. (2020) reported a 97.6% accuracy using the image-based method across 550 samples which were pathologically confirmed [31]. These studies indicate that high sample sizes demonstrate the high accuracy of both methods.

Both approaches were also noted to have high positive and negative predictive values. For the probe-based approach, Kiernan et al. (2021) reported a PPV of 93.0% and an NPV of 100%, while Thomas et al. (2021) reported a PPV of 91.4% and an NPV of 94.1% [28,29]. For the image-based approach, Thomas et al. (2019) found a PPV of 85.7% and an NPV of 82.4%; however, Kose et al. (2020) found a PPV of 95.1% and an NPV of 99.1% [30,31]. The discrepancy between the PPV and NPV of the image-based method is likely due to the difference in sample sizes of the studies; however, these studies still demonstrate that both methods can distinguish PG from non-PG reliably.

Additionally, the sensitivity and specificity were also high for both methods. Using the probe-based method, Thomas et al. (2019) reported a sensitivity of 97.0% and a specificity of 84.2% using the small sample size [30]. Later, Thomas et al. (2021) reported a sensitivity of 95.6% and a specificity of 91.9% [29]. Using the image-based method, Thomas et al. (2019) reported a sensitivity of 90.0% and a specificity of 73.7% [30]. Also using the image-based method, Kose et al. (2020) reported a sensitivity of 98.5% and a specificity of 97.2% while Kim et al. (2018) reported a specificity and sensitivity of 100%, though with a much smaller sample size [27,31].

This study showed that both methods of PG identification are significantly efficient in surgical use and less likely impacted by differences in user technique or use. However, our analysis was limited by the relative paucity of clinical studies that investigated these technologies. The inclusion of only five studies does undermine the statistical power and may undermine the statistical power of the conclusions. Furthermore, limitations do exist in the heterogeneity in the study designs, population, and surgeon expertise that may make the pooled data less reliable. System level limitations in that these technologies require proper and accurate use to obtain accurate data, and the expansion of TP, TN, FP, and FN in these studies. Lastly, Kiernan et al. (2021) did not provide information on the sensitivity and specificity of the probe-based method, which resulted in only Thomas et al. (2021) being used for that assessment [28,29]. Ultimately, three image-based and two probe-based studies were included, but it should be noted that Thomas et al. (2019) used both image-based and probe-based methods [30]. 

Though no meaningful difference can be drawn in comparison at the time of this study, the difference in accuracy could be related to manufacturing rather than to differences in user technique or use. Therefore, if differences do emerge in comparison, this may not reflect the superiority or inferiority of the two technologies but rather the variability in product or prototype manufacturing. However, there is a lack of clinical data comparing the two modalities which may be attributed to the evolving technology and slow commercialization of these instruments within and outside the United States. Thus, additional large-scale studies within institutions are necessary to further compare the modalities. Further topics of discussion and research may include the potential cost savings if these methods can effectively replace pre-operative imaging in parathyroidectomy cases.

## Conclusions

Intraoperative parathyroid preservation during thyroidectomy and identification of parathyroid gland during parathyroidectomy is essential. NIRAF is a non-invasive strategy to identify parathyroid glands intra-operatively. Two identification modalities are available including image and probe-based detection. Both modalities are significantly efficient in surgical use. Though no meaningful difference can be drawn in comparison at the time of this study.
